# Daily Practice and Clinical Expert Teaching

**Published:** 1906-04-07

**Authors:** 


### The Hospital

A Journal of

The Medical Sciences and Hospital Administration.

VOL. XL.—No. 1,019. SATURDAY, APRIL 7, 1906.

### Daily Practice and Clinical Expert Teaching.

In consequence of certain well-known recent developments, it has happened that the term "post-graduation study" has come to have attached to it a somewhat technical and restricted meaning. But in its etymological sense the phrase obviously covers a much wider ground than that included in the schemes of certain colleges and hospitals where the opportunities for study are specially designed for those who already possess a qualification to practise. Equally it may be remarked that, in its literal application, study after graduation is no new development. On the contrary, as has been impressed through numerous academic ages and on successive generations of students, the life of the practitioner of medicine if it is to be effective must be the life of a perpetual student, and certain classical careers, as will readily be recalled, illustrate and emphasise this point. Again, whilst it may be gratefully admitted that some of the existing post-graduation colleges and hospitals offer great and valuable opportunities to those who are able to attend them, they touch, as a matter of fact, but a fraction of the profession; nor is there much reason to believe that this position will be modified in the immediate future.

Evidently, therefore, if the whole field of post-graduation education is to be cultivated and developed, something more than hospital and academic organisation is required. It is allowed that the claims in this direction are, at the present day, and for various causes, more urgent than ever. It is also allowed that for the majority of practitioners the only help which can be provided is that which is brought to their own doors. Whilst all are more or less alive to the increased demands of modern developments, only a small percentage of practitioners can arrange to spend some weeks or months away from the imperious demands of general practice. It is, we think, a more or less conscious recognition of the position thus created that has led to the increased cultivation and publication of medical and surgical problems in the form of the clinical lecture.

The systematic text-book, with its classical description of diseases created by the fusion of numerous individual cases, is a somewhat artificial product, and it is usually neither very easy to read nor very responsive in the presence of particular difficulties. On the other hand, a careful and luminous presentation of the observed facts as these emerge at the bedside in the study of an individual patient, and a commentary on these facts representing the impressions made on the mind of an experienced teacher, find an exact parallel in the daily experience of every practitioner. Such clinical expositions have a strongly pronounced reality and vitality about them, and, if properly digested, form, next to actual personal experience, the most real and confident collection of practical knowledge and power. Hence, this form of record is to be welcomed and encouraged, and it is all to the good that it is receiving wide recognition from some of the most prominent clinical experts. To be really effective, however, the clinical lecture must be worthy of its name. It must not be a mere re-arrangement of the paragraphs of the systematic text-book, and clinical only in the site of its delivery. Rather it must describe and discuss the facts, and the significance of the facts, presented in an actual bedside experience, so that to the reader both the picture of the patient and the effect this produced on the mind of an experienced observer may be presented. Again, the clinical lecture must not be merely a reprint of the patient's journal with its numerous daily details and minutiæ. Rather it must introduce order and perspective among these, and must concern itself less with the mere record of facts and more with the clinical significance which the facts convey. Just as the lawyer cultivates a knowledge of what he calls "case law," so the practitioner should study and preserve a body of clinical records. For practical ends the value of such records can hardly be overstated, and it is therefore with much satisfaction we note that some of our best teachers are applying their energies to this form of post-graduation teaching.

